# Supplementary material for: Environmental modifications of dung beetle larvae shape their growth and life history
Source: J Exp Biol. 2025 Aug 19;228(16):jeb251022. doi: 10.1242/jeb.251022 (PMC12448313; doi:10.1242/jeb.251022)
Supplement: Supplementary information [file jexbio-228-251022-s1.pdf]

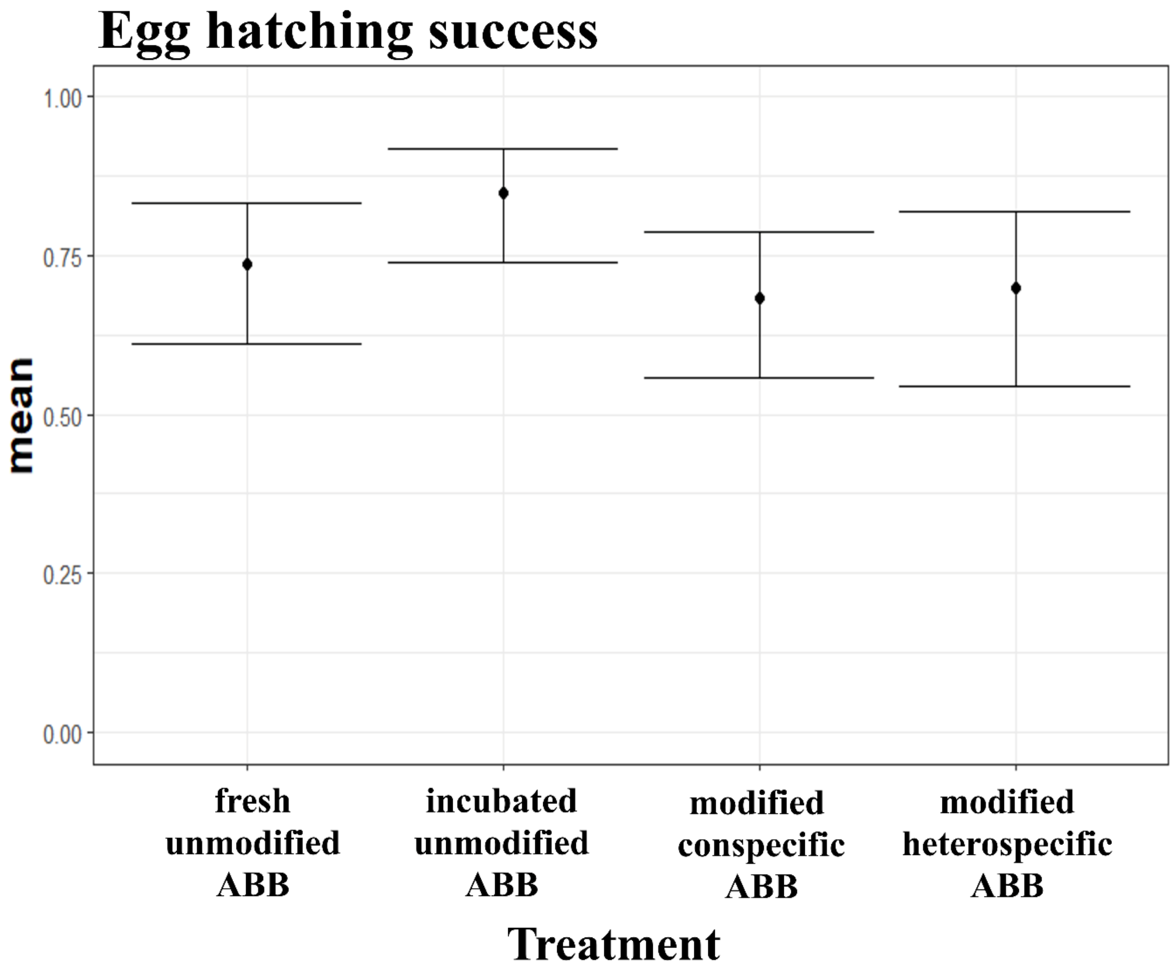

Fig. S1. Mean hatching success for experimental treatments; Fresh Unmodified ABB (n = 60), Incubated Unmodified ABB (n = 57), modified conspecific ABB (n= 60) and modified heterospecific ABB (n = 40). Plots show means (Black dots) and corresponding 95% confidence limits.

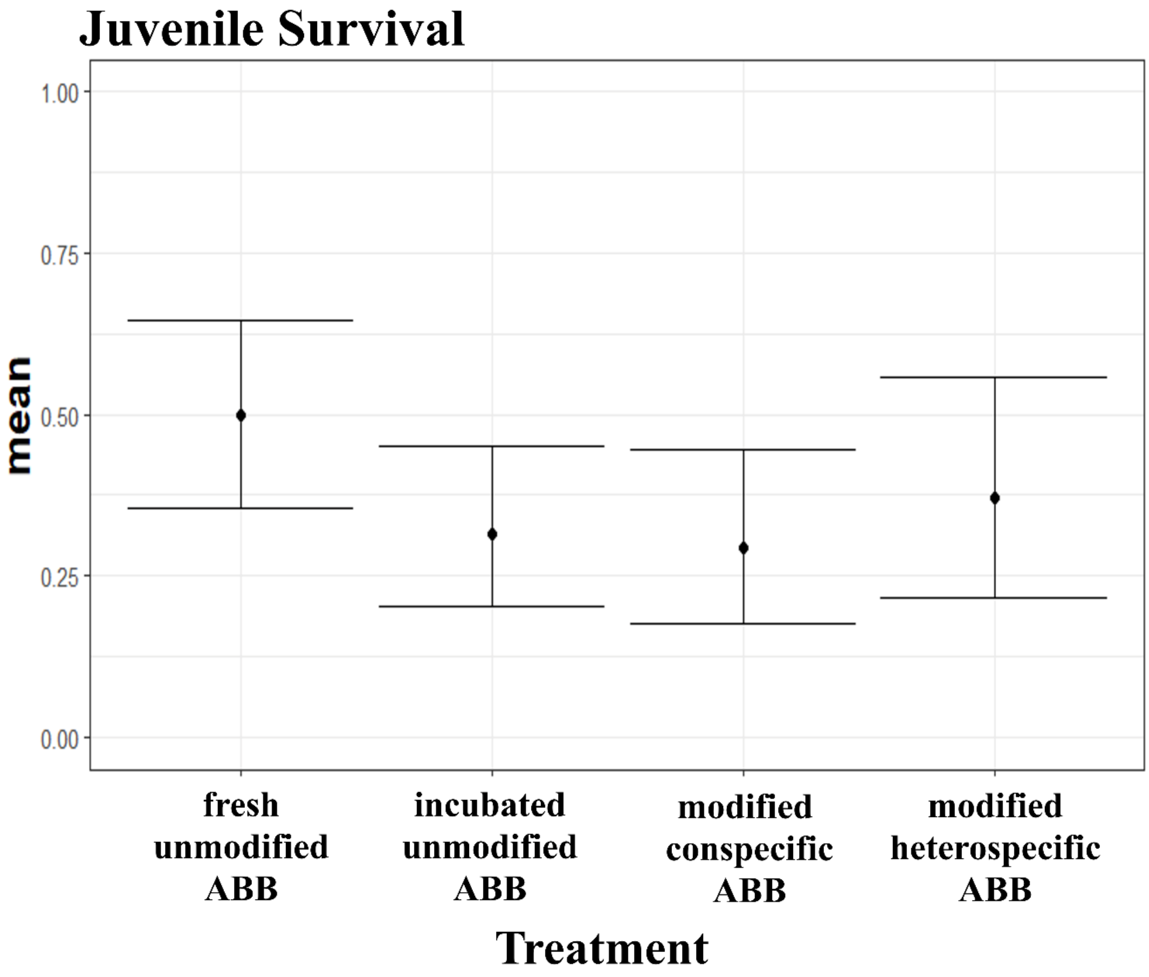

Fig. S2. Mean larval survival for experimental treatments; Fresh unmodified ABB, incubated unmodified ABB, modified conspecific ABB and modified heterospecific ABB. Plots show means (Black dots) and corresponding 95% confidence limits.

Journal of Experimental Biology • Supplementary information
